# BBCA: Improving the scalability of *BEAST using random binning

**DOI:** 10.1186/1471-2164-15-S6-S11

**Published:** 2014-10-17

**Authors:** Théo Zimmermann, Siavash Mirarab, Tandy Warnow

**Affiliations:** 1 Department of Computer Science, University of Texas, Austin, TX, 78712, USA; 2 Département d'Informatique, École Normale Supérieure, Paris, 75005, France; 3 Department of Bioengineering, The University of Illinois, Urbana, IL, 61801, USA

**Keywords:** multi-species coalescent, phylogenomics, incomplete lineage sorting, binning

## Abstract

**Background:**

Species tree estimation can be challenging in the presence of gene tree conflict due to incomplete lineage sorting (ILS), which can occur when the time between speciation events is short relative to the population size. Of the many methods that have been developed to estimate species trees in the presence of ILS, *BEAST, a Bayesian method that co-estimates the species tree and gene trees given sequence alignments on multiple loci, has generally been shown to have the best accuracy. However, *BEAST is extremely computationally intensive so that it cannot be used with large numbers of loci; hence, *BEAST is not suitable for genome-scale analyses.

**Results:**

We present BBCA (boosted binned coalescent-based analysis), a method that can be used with *BEAST (and other such co-estimation methods) to improve scalability. BBCA partitions the loci randomly into subsets, uses *BEAST on each subset to co-estimate the gene trees and species tree for the subset, and then combines the newly estimated gene trees together using MP-EST, a popular coalescent-based summary method. We compare time-restricted versions of BBCA and *BEAST on simulated datasets, and show that BBCA is at least as accurate as *BEAST, and achieves better convergence rates for large numbers of loci.

**Conclusions:**

Phylogenomic analysis using *BEAST is currently limited to datasets with a small number of loci, and analyses with even just 100 loci can be computationally challenging. BBCA uses a very simple divide-and-conquer approach that makes it possible to use *BEAST on datasets containing hundreds of loci. This study shows that BBCA provides excellent accuracy and is highly scalable.

## Background

Species tree estimation from multiple loci is complicated by incomplete lineage sorting (ILS), a population-level process that produces gene trees that differ from each other and from the true species tree [1]. Furthermore, when ILS levels are sufficiently high, the standard approach of concatenating alignments for each locus together into a larger supermatrix and then estimating the tree from the supermatrix (for example, using maximum likelihood) can produce incorrect trees with high confidence [2]. Because concatenated analyses can be positively misleading and even the most frequently observed gene tree topology can be different from the species tree in the presence of ILS [3], coalescent-based methods for multi-locus species tree estimation have been developed [4,5].

Here we address the challenge of using *BEAST and other Bayesian coalescent-based methods for co-estimating species trees and gene trees. These methods are statistically consistent under the multi-species coalescent model [6], which means that as the number of genes and their sequence lengths both increase, the probability that the method will return the true species tree will increase to 1. While these Bayesian methods have excellent accuracy in simulations and on biological datasets [7-9], they use computationally intensive MCMC approaches that in practice limit them to relatively small numbers of loci; for example, *BEAST did not converge on 100-gene simulated datasets with 11 taxa within 150 hours [9], and analyses on biological datasets can take weeks [10].

Alternative coalescent-based techniques operate by combining estimated gene trees, of which MP-EST [11] is among the most popular. Some of these "summary methods" (e.g., STAR [12], STEM [13], BUCKy-pop [14], ASTRAL [15], and MP-EST) are statistically consistent in the presence of ILS, and are far less complicated to use than *BEAST or other fully parametric methods [9,16]. Furthermore, some of these summary methods are quite fast and can analyze datasets with 100 or more loci without any difficulty [16-18]. Thus, for computational reasons many multi-locus phylogenomic datasets are analyzed using summary methods [17,18]. However, co-estimation methods, such as *BEAST, are generally preferred over summary methods, and even the most popular and best performing summary methods (e.g., MP-EST) have been criticized by some biologists as being unsatisfactory "short-cut" methods [19]. Thus, enabling fully parametric methods such as *BEAST to be used on phylogenomic datasets with hundreds or thousands of loci is an important objective.

## Approach

### BBCA: Boosted Binned Coalescent Analysis

As shown in [9], gene trees estimated by *BEAST can be much more accurate than trees estimated using RAxML [20] or FastTree [21] maximum likelihood, with the biggest improvements occuring when there are low levels of ILS and sequence lengths are not very long. When *BEAST produces more accurate gene trees, it also produces more accurate species trees than coalescent-based summary methods applied to gene trees estimated by maximum likelihood methods. Furthermore, applying summary methods (such as MP-EST) to the *BEAST gene trees produced species tree estimations that were as accurate as *BEAST, suggesting that the main advantage *BEAST provided over summary methods was due to its ability to produce more accurate estimated gene trees [9].

These observations motivate the design of BBCA (Boosted Binned Coalescent Analysis), our proposed pipeline for coalescent-based species tree estimation. BBCA takes as input a set of sequence alignments for a set *S *of species, and then performs the following three steps:

• Step 1: Randomly partition the loci into bins of approximately the same size (where the number of bins is chosen by the user).

• Step 2: For each bin, run *BEAST on the set of multiple sequence alignments in each bin to co-estimate the gene trees and species tree for the bin.

• Step 3: Run MP-EST on the set of estimated gene trees, to produce an estimated species tree called the BBCA tree.

This technique has some similarities to the "Naive Binning" method [9], but differs in important ways that change its statistical properties. Naive binning performs the same random partitioning, but then concatenates the sequence alignments within each bin and computes a "supergene" tree on the bin using concatenation. Finally, the supergene trees are combined using a summary method. Naive Binning lacks a theoretical basis, since there is no attempt to ensure that genes in the same bin have the same tree; however, as shown in [9], this technique improved the accuracy of MP-EST and other coalescent-based methods. The technique we propose here, however, does have a theoretical basis, because *BEAST is used to estimate the gene trees within each bin, and these gene trees are then combined using a statistically consistent coalescent-based method. Thus, BBCA is statistically consistent under the multi-species coalescent when the size of the bins grows with the number of genes. (It is, however, an open question as to whether BBCA is statistically consistent with constant bin size.)

*Variants on this default setting for BBCA*. We show results for BBCA where we set the number of genes per bin to 25 and otherwise follow the default settings shown above. A simple variant of this pipeline would change the number of genes per bin, but other variants can also be considered. For example, a different co-estimation method (e.g., BEST [22]) could be used to estimate the gene trees, and a different coalescent-based summary method (e.g., ASTRAL [15]) could be used to combine the estimated gene trees into a species tree. Another alternative to this pipeline would use *BEAST to produce a distribution of gene trees for each locus, and then apply the summary method to the combined set of distributions of gene trees. Results for some variants of the protocol are shown in the supplementary materials, and summarized below.

### Datasets and computational platform

We explore *BEAST and BBCA using two collections of simulated datasets. All BBCA analyses were run on the Texas Advanced Computing Center (TACC), with most on the Lonestar cluster, which limits analyses to 24 hours, and some also on the Stampede cluster, which allows longer analyses. All *BEAST analyses were run on TACC's Stampede cluster. Each *BEAST analysis on each bin of 25 genes within BBCA was limited to either 24 or 48 hours. We also performed additional *BEAST analyses in which we explored longer (96- or 168-hour) runs on 100-gene datasets. After *BEAST completed on each bin, we ran MP-EST 10 times using the maximum clade credibility tree output by *BEAST for each gene, and kept the tree with the best pseudo-likelihood score.

*Simulated datasets*. We had two types of simulated datasets - 11-taxon "strongILS" datasets originally studied in [23] (and later used in [9]) and 12-taxon "Laurasiatheria" datasets that we generated for this study based on a coalescent-based analysis of the mammalian phylogeny in [17]. Both collections were obtained by generating a species tree, evolving 100 gene trees down the species tree under the multispecies coalescent model, and then evolving sequences down each tree under a GTR (General Time Reversible) sequence evolution model. Each model condition has 10 replicates. These datasets have outgroups, so that MP-EST analyses (which require rooted gene trees) can be performed.

The 11-taxon strongILS datasets all have the same model species tree, which has relatively high levels of ILS. Gene trees were simulated down the model species tree, and then sequences were evolved down the different gene trees under very heterogeneous models of evolution. Each sequence dataset has length 500, and so the alignments are relatively short.

To produce the simulated Laurasiatheria datasets, we computed an MP-EST tree on a dataset of 424 loci for a 37-species mammalian dataset originally studied in [17]. We restricted this tree (with its branch lengths in coalescent units) to a subset of 11 Laurasitheria species and one outgroup species (homo sapiens), to produce the model species tree for this simulation. We evolved gene trees down the model tree under the multi-species coalescent using Dendropy [24], and then evolved sequences down each gene tree using bppseqgen [25]. We varied the length of the sequences from 500 bp up to 1500 bp.

### Analyses performed

We used RAxML and FastTree to compute gene trees on the gene sequence alignments, and RAxML to compute concatenation analyses using maximum likelihood (CA-ML). We used *BEAST and BBCA to compute gene trees and species trees on the multi-marker datasets.

### Metrics

We report average gene tree and species tree estimation error using the normalized Robinson-Foulds [26] distance to the true (model) gene tree and species tree, respectively. We assessed convergence using the first seven effective sample size (ESS) values provided in the report created by treeannotator (a tool provided in *BEAST); according to the *BEAST instructions, these values should be above 100 for the *BEAST run to be considered to have converged. We report a sample of these ESS values, as well as the number of MCMC iterations, running time, and peak memory usage for each analysis.

## Results

We present results for these analyses here, but see Additional file 1 for additional details.

**Figure 1:**
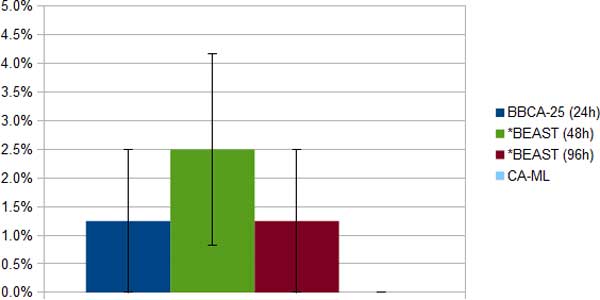
Average species tree estimation error (and standard error bars) on the ten 11-taxon 100-gene strongILS simulated datasets using BBCA (with 24-hour *BEAST analyses on each 25-gene bin), *BEAST (with a 48- or 96-hour runtime limit) and concatenation using RAxML.

*11-taxon strongILS datasets*. The 11-taxon datasets have 100 genes, and so each dataset was analyzed using BBCA on four bins of size 25, with *BEAST run for 24 hours on each bin. We also ran *BEAST on the full set of 100 genes for 48 and 96 hours. We compare the topological error of species trees computed using three methods: BBCA, *BEAST, and concatenation using maximum likelihood (CA-ML) (Figure 1). Concatenation had perfect accuracy (no error on any dataset), and BBCA and the 96-hour *BEAST analysis both had the same excellent performance (1.25% tree error, indicating one incorrect branch in one out of ten trees). The 48-hour *BEAST analysis had double the error of the BBCA and 96-hour *BEAST analysis.

**Figure 2:**
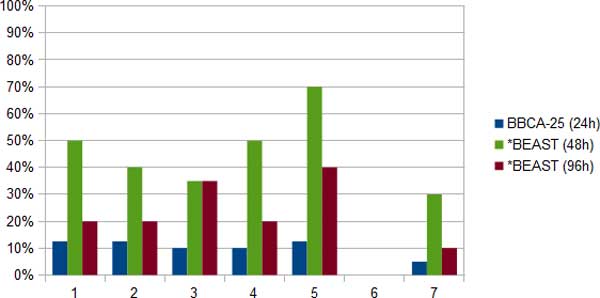
**Proportion of ESS values below the minimum threshold (100) for convergence for the ten 11-taxon strongILS datasets**. We show results when running BBCA (using 24 hours per 25-gene bin, in blue), *BEAST (using 48 hours on the sets of 100 genes, in green) or *BEAST (using 96 hours, in dark red). We report these proportions for seven different statistics: (1) posterior, (2) prior, (3) likelihood, (4) species.coalescent, (5) species.popSizesLikelihood, (6) speciation.likelihood, (7) species.popMean. Thus, BBCA has converged for 85-95% of the runs, using 24 hours per bin. In contrast, *BEAST has converged for only 60-90% of the runs after running for 96 hours.

The ESS values suggest that *BEAST was much closer to converging when run in the BBCA analysis than when run on the full set of 100 genes, even when *BEAST was allowed to run for 96 hours (Figure 2). Peak memory usage was also greatest for the 96-hour *BEAST analysis (665 Mb), and least for the BBCA analysis (448 Mb). The average number of MCMC iterations reached by BBCA within each bin was 158 million, while the 48-hour *BEAST analysis reached 204 million, and the 96-hour *BEAST analysis reached 479 million.

**Figure 3:**
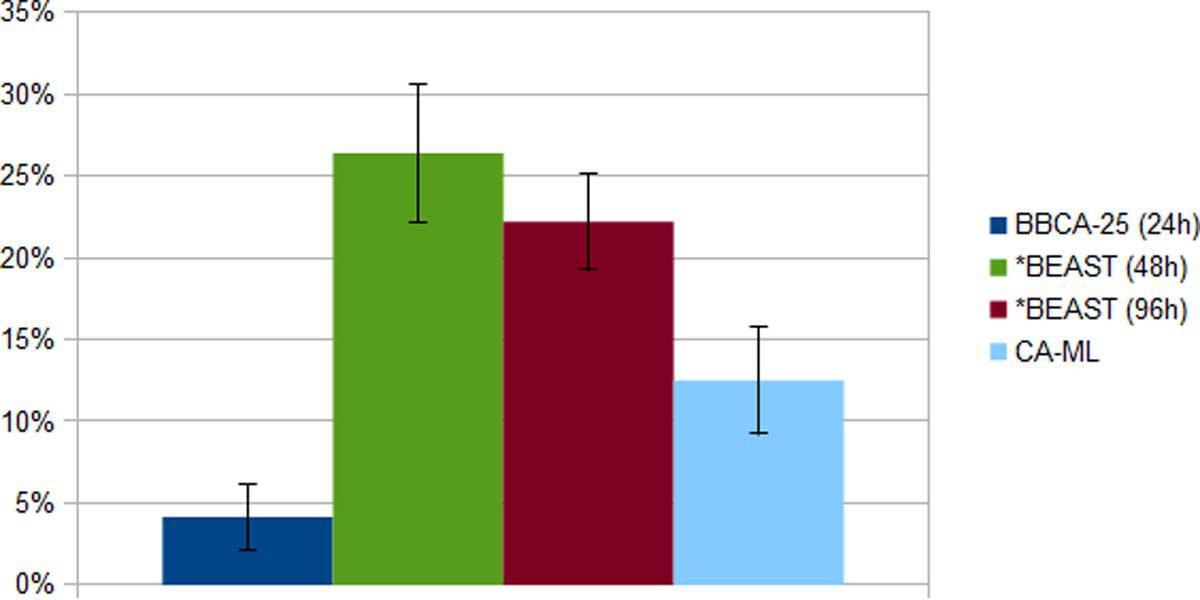
Average species tree estimation error (and standard error bars) on eight Laurasiatheria 1500 bp simulated datasets using BBCA, *BEAST and concatenation; BBCA is run with a 24-hour time limit on each 25-gene bin, and *BEAST is run with a 48-hour or 96-hour time limit. Increasing the time per bin to 48 hours did not change the accuracy for BBCA.

**Figure 4:**
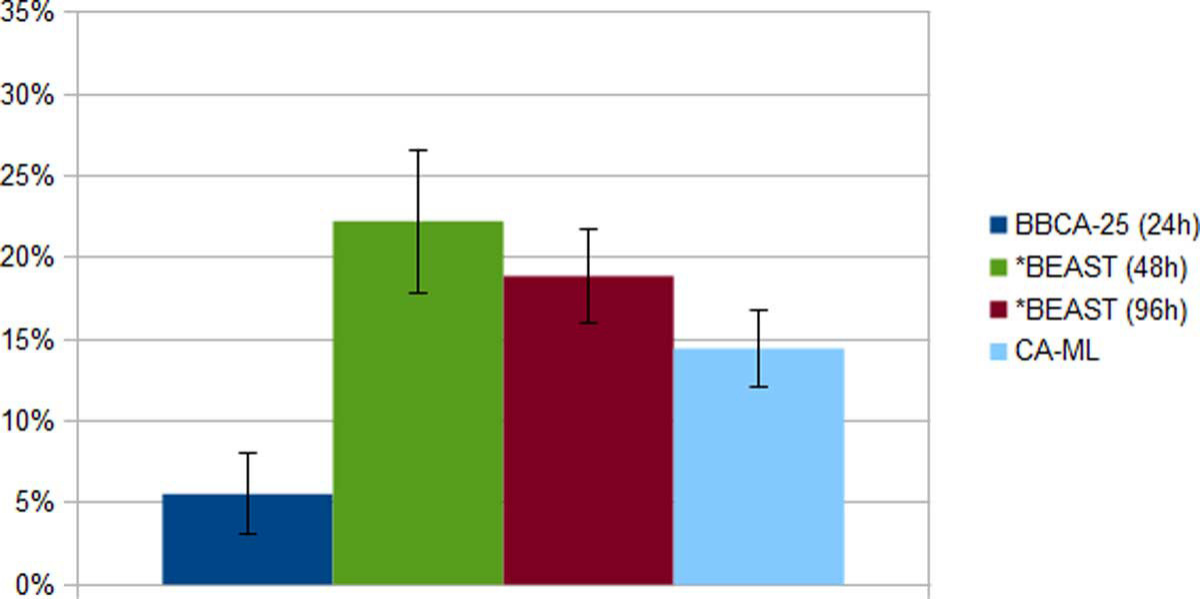
Average species tree estimation error (and standard error bars) on the ten Laurasiatheria 1000 bp simulated datasets using BBCA, *BEAST and concatenation; BBCA is run with a 24-hour time limit on each 25-gene bin, and *BEAST is run with a 48-hour or 96-hour time limit.

**Figure 5:**
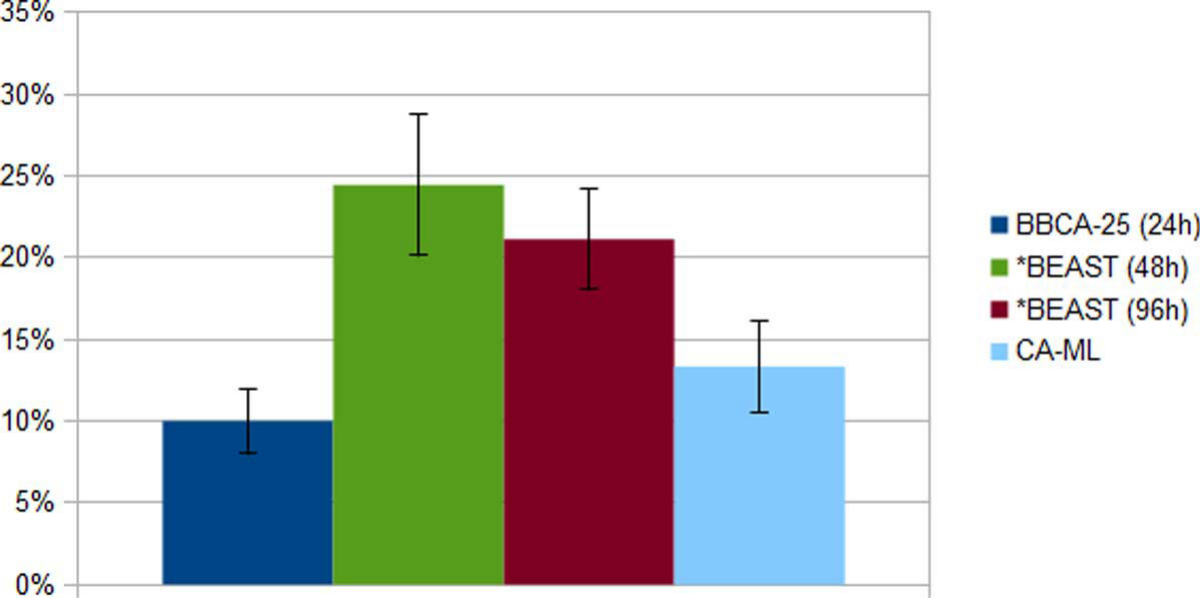
Average species tree estimation error (and standard error bars) on the ten Laurasiatheria 500 bp simulated datasets using BBCA, *BEAST and concatenation; BBCA is run with a 24-hour time limit on each 25-gene bin, and *BEAST is run with a 48-hour or 96-hour time limit.

*Laurasiatheria simulated datasets*. We compared BBCA, *BEAST, and CA-ML on the Laurasitheria simulated datasets, using 24-hour analyses of *BEAST on each bin of size 25, and allowing up to 96 hours for *BEAST on the 100-gene datasets. *BEAST failed to run on two of the 1500 bp datasets; consequently, all our comparisons for the 1500 bp datasets are based on the eight remaining replicates. Results for the CA-ML, BBCA, and the 48- and 96-hour *BEAST analyses are shown in Figures 3, 4 and 5. The 96-hour *BEAST analysis was more accurate than the 48-hour *BEAST analysis, but even the 96-hour analysis was *much less *accurate than the BBCA analysis. Interestingly, for all these conditions, BBCA was more accurate than CA-ML, even though CA-ML was more accurate than the 96-hour *BEAST analysis.

The first seven ESS values suggest that BBCA may have converged on these data (all values except one are above 100), while even the 96-hour *BEAST analysis was far from converging for many of the statistics (see supplementary materials for additional results and discussion).

From a computational perspective, BBCA used less peak memory than the 48- hour and 96-hour *BEAST analyses: BBCA used from 440 Mb-487 Mb of peak memory, and the *BEAST analyses used from 891 Mb to 999 Mb.

**Figure 6:**
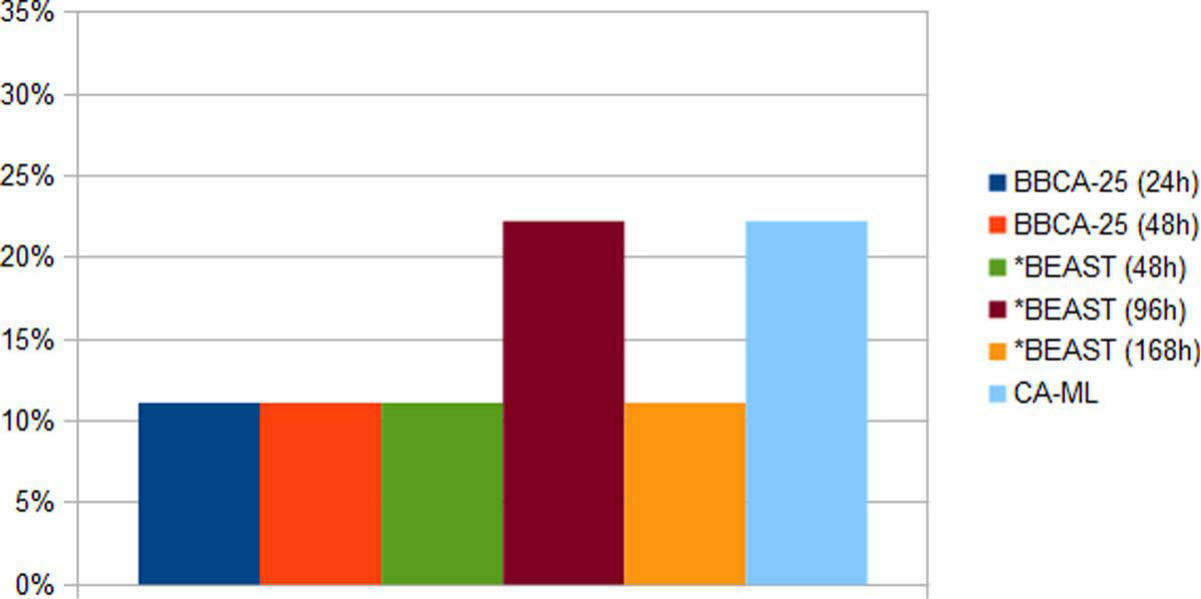
Average species tree estimation error on one Laurasiatheria 1500 bp simulated dataset using BBCA, *BEAST and concatenation; BBCA is run with a 24-hour or 48-hour time limit on each 25-gene bin, and *BEAST is run with a 48-hour, 96-hour or 168-hour time limit. Increasing the time limit for *BEAST to 168 hours did not allow it to have better accuracy than BBCA.

**Figure 7:**
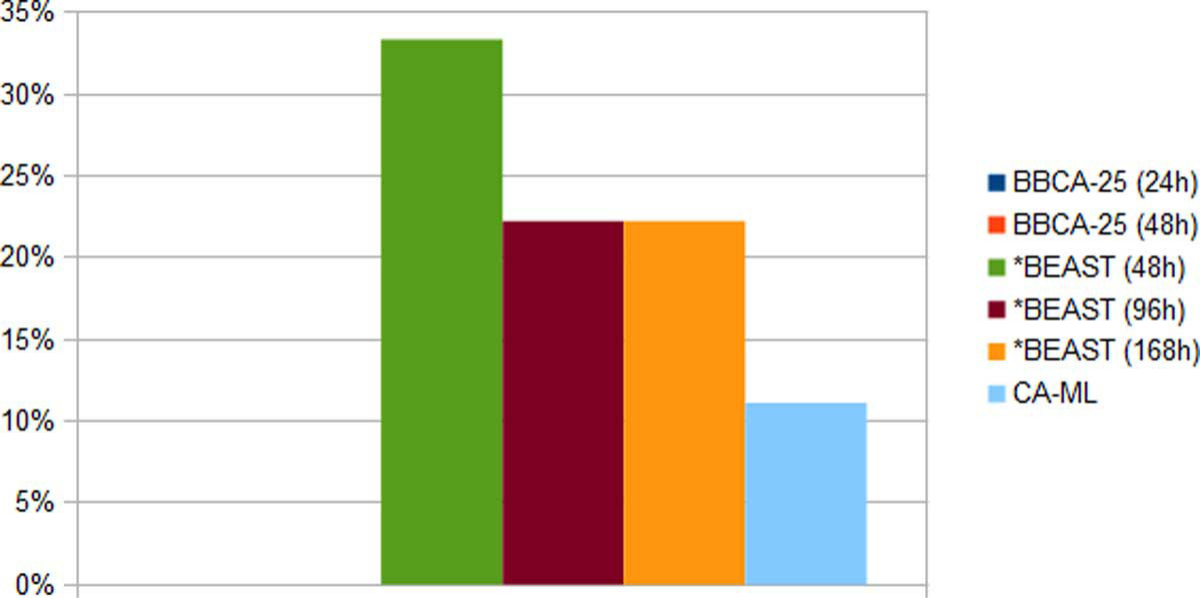
Average species tree estimation error on one Laurasiatheria 1000bp simulated dataset using BBCA, *BEAST and concatenation with maximum likelihood (CA-ML). BBCA is run with a 24-hour or 48-hour time limit on each 25-gene bin, and *BEAST is run with a 48-hour, 96-hour or 168-hour time limit. BBCA using either 24 hours or 48 hours per bin recovers the true species tree, but both *BEAST analyses and the CA-ML fail to recover the true species tree.

Week-long *BEAST analyses. We ran two week-long (168-hour) long *BEAST analyses, each on one replicate of two model conditions of the Laurasiatheria simulated datasets - one with a 1500 bp alignment (Figure 6) and the other with a 1000 bp alignment (Figure 7). On the 1000 bp alignment, BBCA (run with 24 or 48 hours on each of the four 25-gene bins) had perfect accuracy. In contrast, the week-long *BEAST analysis on the 1000 bp alignment had 22% error, and the 48-hour *BEAST analysis had 33% error. CA-ML was much more accurate than the *BEAST analyses on the 1000 bp dataset (with about half the error of the 168-hour *BEAST analysis), but was less accurate than BBCA.

On the 1500 bp alignment, BBCA using 24 hours on each bin matched the accuracy of the week-long *BEAST analysis (11% error). CA-ML had 22% error on the 1500 bp dataset, and so was not as accurate as BBCA or 168-hour *BEAST, although it matched the 96-hour *BEAST analysis.

Since convergence may have been an issue, we examined the ESS values for these analyses (see Tables 1 and 2). On the 1000 bp alignment, *BEAST seemed to have converged for most statistics in 48 hours, but the ESS values were much better on the 168-hour analysis (all were above 100, all but one were above 750, and most were above 14,000). Thus, on this dataset the 168-hour *BEAST analysis seemed to have converged, and yet CA-ML was still more accurate than *BEAST. On the 1500 bp alignment, *BEAST had clearly failed to converge in 48 hours (except for one statistic) but had excellent ESS values after 168 hours. BBCA matched or improved on concatenation on all these model conditions.

**Table 1:** ESS values when running *BEAST for 48, 96 or 168 hours on one Laurasiatheria dataset with 1500 bp.

Statistics	*BEAST (48 h)	*BEAST (96 h)	*BEAST (168 h)
posterior	93	5424	12813
prior	55	5517	14265
likelihood	12	72	184
species.coalescent	77	5635	14320
species.popSizesLikelihood	9	47	1303
speciation.likelihood	2112	5053	4722
species.popMean	11	29	1679

*BEAST reached an average of 92 million iterations in 48 hours, an average of 257 million iterations in 96 hours and an average of 597 million iterations in 168 hours.

**Table 2:** ESS values when running *BEAST for 48, 96 or 168 hours on one Laurasiatheria dataset with 1000 bp.

Statistics	*BEAST (48 h)	*BEAST (96 h)	*BEAST (168 h)
posterior	2128	5966	15274
prior	2192	6948	15162
likelihood	175	150	154
species.coalescent	2139	6966	15446
species.popSizesLikelihood	7	13	761
speciation.likelihood	2248	6989	14804
species.popMean	13	24	1109

*BEAST reached an average of 114 million iterations in 48 hours, an average of 301 million iterations in 96 hours and an average of 656 million iterations in 168 hours.

### Results on BBCA variants

*Bin size*. We compared BBCA analyses using bins of size 50 and allowing 48 hour *BEAST analyses of each bin, to the default case of bins of size 25 and allowing 24 hour *BEAST analyses of each bin. On the simulated Laurasiatheria datasets with 1000 bp, the BBCA analysis with bins of size 50 produced species trees with 7.8% tree error, but using BBCA on bins of size 25 produced species trees with 5.6% tree error. Thus, when the *BEAST analyses of all the bins together were limited to a total of 96 hours, the smaller bin size produced better results.

The ESS values on the bins of size 50 for *BEAST analyses using 48 hours were about the same as those for the 24-hour *BEAST analyses of the bins of size 25. Although the ESS values suggest that both analyses had reached comparable levels of convergence, the difference in species tree accuracy may reflect a failure of *BEAST to converge sufficiently on the 50-gene bins. Put differently, the way we report ESS values may not indicate perfectly the level of convergence for *BEAST analyses.

Using *BEAST to produce a distribution of trees for each gene. *BEAST is a Bayesian method that uses MCMC to co-estimate the gene trees and species tree given a set of multiple sequence alignments on different loci. Thus, it can be used to produce a distribution of gene trees (one distribution for each gene) as well as a single gene tree (the maximum clade credibility tree) for each gene. In the default version of BBCA, we applied MP-EST to the set of maximum clade credibility gene trees to obtain an estimated species tree; however, MP-EST could also have been applied to a set consisting of the union of the distributions of gene trees produced by *BEAST on each gene. We explored the impact of using the distribution of gene trees instead of a single gene tree on the simulated datasets.

Results on the simulated Laurasiatheria datasets allowed us to examine the impact using different sequence lengths as well. For the longest sequences (1500 bp), the species tree estimation error using maximum clade credibility gene trees or using 100 gene trees (sampled from the *BEAST distribution) for each gene was the same - 4.2% tree error. For the medium length sequences (1000 bp), using the maximum clade credibility gene trees produced a less accurate tree (5.6% tree error) than using the distribution of gene trees (3.3% tree error). For the shortest sequences (500 bp), there was no difference in accuracy between the two ways of running BBCA (10% tree error whether based on maximum clade credibility gene trees or based on 100 sampled gene trees per gene).

We examined the average gene tree estimation error for the single maximum clade credibility gene tree per locus as opposed to the average gene tree error in the distributions produced by *BEAST. We observed that the average gene tree estimation error differed between the two ways of using *BEAST but depended on the sequence length; thus, on 1500 bp alignments, the average gene tree estimation error for the maximum clade credibility gene trees was 28.0%, and when based on the distribution was 30.0%; on 1000 bp alignments the average gene tree estimation error was 29.9% when based on maximum clade credibility gene trees and was 32.5% when based on the distribution of gene trees; and on the 500 bp alignments the gene tree estimation error was 32.9% when based on the maximum clade credibility gene trees and 37.3% when based on the distribution of gene trees. Thus, at least for these data, using the distribution of gene trees was generally benign - either not changing the result or reducing the error.

Increasing runtimes for *BEAST within BBCA. We examined the impact of allowing *BEAST to run for 48 hours instead of 24 hours on each 25-gene bin on the Laurasiatheria simulated datasets. ESS values improved for *BEAST on these bins when run for 48 hours, but there was no change in the resultant species tree estimation for any sequence length condition, whether we ran *BEAST for 24 hours or 48 hours for each bin.

### Computational issues

The BBCA pipeline has three steps: divide loci into bins, run *BEAST on each bin, and then combine gene trees using MP-EST. The first step is trivially fast. The second second step is the most computationally intensive, with the running time per bin depending on the user, but needing to be at least 24 hours (and more, for larger numbers of genes or higher rates of ILS). The running time for the third step (MP-EST) depends on the number of species more than the number of genes, and so is fast for the datasets that are suitable for *BEAST.

MP-EST is a heuristic for a "pseudo-maximum likelihood" optimization problem, which is based on the distribution of rooted three-taxon gene trees defined by the input gene trees. MP-EST operates in two steps, where the first step computes this distribution (and is impacted by the number of genes, but is very fast) and the second step searches for the "pseudo-maximum likelihood" solution, and is slower. Hence, the number of loci has a very small impact on MP-EST's running time, since it only impacts the initial part of the analysis. The number of species impacts MP-EST's running time [27], but for small numbers of taxa (as in this study), MP-EST is very fast. On our datasets, MP-EST always completed in less than 2 minutes.

## Discussion

Although this study was limited to two basic model conditions, the results show that a simple way of running the BBCA pipeline produced trees that matched or improved on the accuracy of *BEAST, when both were run in a time-limited fashion. This is not surprising, since BBCA's partitioning step makes it more likely that *BEAST will converge on each bin, and thus produce highly accurate gene trees. Since *BEAST's accuracy as a species tree method seems largely a function of the accuracy of its gene trees, BBCA is more accurate than *BEAST under conditions where *BEAST can converge well on the bins but does not converge well on the full set of loci. Thus, BBCA was not designed for use on datasets where *BEAST can converge well (i.e. with small enough numbers of loci), and on such datasets we do not expect to see improvements on results obtained by *BEAST. On the other hand, we expect BBCA to be more accurate than *BEAST on datasets with large numbers of loci where running *BEAST to convergence is difficult or infeasible.

Given this, the observation that BBCA is sometimes more accurate than *BEAST, and yet ESS values suggest that *BEAST had reached convergence, is surprising. One possible explanation is that ESS values may need to be much higher than 100 (the minimum required) to be reliable indications of convergence. If so, then the time needed for *BEAST to converge may be even larger than the earlier estimates suggested (which were based on ESS values needing to be above 100). Another possibility is that there are other ESS values that are more predictive of species tree topology accuracy and that we did not examine, and which were below 100 in these analyses.

However, practical issues made it infeasible to report all of the ESS values, since on datasets with 100 genes there were as many as 1444 statistics whose ESS values could be computed. In our study it would have been impossible to make sure all the replicates were above these limits, even with the smallest bins.

The study also showed conditions in which the gene trees estimated by *BEAST were less accurate than ML gene trees estimated using RAxML or FastTree; whether this is due to a failure to converge or some other issue is unknown. A direct comparison to summary methods applied to ML gene trees would be very interesting, and might reveal conditions in which summary methods can outperform *BEAST.

The study also suggested the possibility that variants of the BBCA pipeline might produce further improvements; for example, using *BEAST to produce a distribution of trees for each gene rather than a single point estimate gave a small advantage for one condition and was otherwise neutral, and so additional study of this variant would be desirable.

Future studies should examine the impact of using binning under other model conditions, including missing data (gene sequence alignments that do not include all the species), different amounts of ILS, and in the presence of other sources of gene tree discord (such as gene duplication and loss or horizontal gene transfer).

It would also be very interesting to explore the impact of binning on branch length estimation, since branch lengths (in coalescent units) are parameters that are estimated by coalescent-based methods, such as *BEAST and MP-EST, and are used to estimate the amount of ILS in the data or to date ancestral nodes.

The analyses we performed used MP-EST to combine the estimated gene trees, but BBCA could be used with other coalescent-based summary methods and would still be statistically consistent under the coalescent model. However, the empirical performance could change, and the use of summary methods such as ASTRAL or BUCKy-pop would make it feasible to run BBCA without reliable outgroups.

Increasing the bin size could improve the gene tree accuracy but might entail a running time increase, to ensure convergence in each bin. Indeed, our study suggested that 24-hour analyses were sufficient on the 25-gene datasets, but that even 48-hour analyses were perhaps insufficient on the 50-gene datasets we examined. However, these analyses were under high levels of ILS, and it is possible that 24-hour analyses of larger bins might be feasible if the amount of ILS were lower. Thus, increasing the bin size might be best for accuracy, but the computational feasibility of larger bins will likely depend on various properties of the data, such as amount of ILS, sequence length, branch lengths in the gene trees, and phylogenetic signal in the sequence alignment. However, generally not much is known about the running time and memory requirements of *BEAST, and how the dataset properties impact these requirements, and so answering these questions will require substantial exploration and study.

Non-random binning might have advantages in terms of running time and accuracy, and could be explored. For example, if genes were binned based on shared GTR model parameters (e.g., the substitution matrix), *BEAST might converge more quickly on each bin, produce more accurate gene trees, and result in more accurate species trees. Empirical performance therefore might be improved, even if the basis for the guarantee of statistical consistency (which requires random sampling of genes) was weakened by non-random binning.

Because MP-EST is extremely fast (running typically in minutes, even for thousands of loci), the running time is largely a function of the requirement to run *BEAST on each bin. These *BEAST analyses of different bins are entirely independent, and so can be run in parallel; hence, BBCA is very scalable, and is likely to exhibit near-linear scalability with the number of processors. For example, a dataset with 1000 loci could be analyzed using 40 processors, each running *BEAST on 25 loci, and the results could then be combined in minutes using MP-EST. Thus, BBCA enables *BEAST, a sophisticated Bayesian method for co-estimating the species tree and gene trees under the multi-species coalescent model, to be used on genome-scale datasets.

Finally, BBCA addresses computational limitations to small numbers of genes, but does not address the limit of *BEAST to small numbers of species (currently perhaps in the 20's). Thus, one of the outstanding problems is how to improve *BEAST's scalability so that it can analyze datasets with 50 or 100 species in reasonable timeframes. Divide-and-conquer strategies are a possibility for improving the scalability of phylogenomic analyses from multiple markers, and have been studied in the context of MP-EST [27] and shown to maintain or improve accuracy.

## Conclusions

*BEAST is one of the few fully-parametric methods that is used in biological dataset analyses to provide a coalescent-based species tree estimation [10]. However, *BEAST is computationally too intensive for most users to run it successfully on datasets with 100 or more loci. For this reason, *BEAST and other co-estimation methods have been considered inapplicable to phylogenomic datasets, where hundreds to thousands of loci are sampled from throughout the genome [8], and most biological datasets (except very small ones) are analyzed instead using summary methods. Yet, some biologists find summary methods unsatisfying compared to *BEAST and other fully parametric methods, resulting in dissatisfaction with existing coalescent-based methods [19].

BBCA was designed to address the computational challenge in using *BEAST on datasets with many loci. The design is very simple: random partitioning of the genes into small bins (containing only 25 genes), running *BEAST on each bin to estimate gene trees, and then estimating the species tree from the gene trees using MP-EST. Furthermore, although we only explored performance on two model conditions and only for a small number of ways of running BBCA, this study showed that BBCA analyses produced highly accurate species trees that matched or improved on the accuracy of much longer *BEAST analyses. By design, BBCA can be run on very large number of genes, and thus improves the scalability of *BEAST.

## Methods

*BEAST. We used a command-line script called "create_beast_input.pl" (which we are making publicly available, see the Availability of datasets section) to produce *BEAST input files. We fixed the number of MCMC iterations to 1 billion (a number that was too large to be ever reached in our time-limited analyses) and the sample rate to 40,000 (so that the distributions and log files produced would have between 1,000 and 10,000 lines in practice).

To call *BEAST, we used the command:

beast -warnings -strict -working -overwrite -errors 0

-threads 2 -seed 1234 input.xml

We used *BEAST on two different types of machine, one running version 1.7.4 and the other running version 1.7.5. The older version of *BEAST was only used within BBCA (24-hour analyses), and the newer version was used for all the remaining analyses (in particular when we ran *BEAST on the full set of genes). According to the version history provided, changes between the versions consist for the most part in improvement to BEAUti, a piece of software that we did not use, or new options in *BEAST. Some minor issues were also fixed but they are not likely to have affected our results (and to the extent they might have, the newer version would produce better results). We always ran two independent instances of *BEAST on the same input and to do so, we provided different random seeds.

For each gene or species tree, we obtained distributions from the two instances of *BEAST and discarded the first 50% of each distribution as "burn-in". Then, we combined the two distributions together and computed the maximum clade credibility gene tree by using the following command:

treeannotator -heights mean -burnin 0 input_distribution

output_tree

We also randomly subsampled 100 trees from each distribution for the variant where MP-EST is applied to multiple gene trees for each locus. We similarly removed burn-in from the log files of *BEAST and combined them before using loganalyzer, which is a piece of software which comes with *BEAST, to compute the ESS values. The command was:

loganalyser -burnin 0 input_log output_file

*MP-EST*. We combined maximum clade credibility gene trees or distributions of gene trees with MP-EST version 1.2. When combining distributions of gene trees we provided the 100 sub-sampled trees for all genes as one large input to MP-EST.

*RAxML and FastTree ML tree estimation*. RAxML version 7.9.4 was used to compute ML gene trees with the command:

raxmlHPC-PTHREADS-SSE3 -T 2 -m GTRGAMMA -s input_sequence

-n output_id -N 20 -p 1234

FastTree verson 2.1.7 SSE3 was used to compute ML gene trees with the command:

FastTree -nt -gtr < input_sequence > output_tree

## Competing interests

The authors declare that they have no competing interests.

## Authors' contributions

TW conceived the study and drafted the manuscript; SM provided the simulated data and helped write the manuscript; and TZ performed the analyses and helped write the manuscript. All authors read and approved the final manuscript.

## Availability of datasets

The Laurasiatheria dataset is new and so we make it publicly available. We also make publicly available all the *BEAST input files we produced and the script that was used to produce them. All these can be found at http://www.cs.utexas.edu/users/phylo/datasets/bbca/.

## Supplementary Material

Additional file 1 1471-2164-15-S6-S11-S1.pdf. Additional figures and tables omitted from the main paper due to space constraints are presented here. Click here for file

## Declarations

This work was partially supported by the National Science Foundation grants DEB 0733029 and DBI 1062335 to TW and by the Howard Hughes Medical Institute to SM.

This article has been published as part of *BMC Genomics *Volume 15 Supplement 6, 2014: Proceedings of the Twelfth Annual Research in Computational Molecular Biology (RECOMB) Satellite Workshop on Comparative Genomics. The full contents of the supplement are available online at http://www.biomedcentral.com/bmcgenomics/supplements/15/S6.
