# Supplementary material for: BBCA: Improving the scalability of *BEAST using random binning
Source: BMC Genomics. 2014 Oct 17;15(Suppl 6):S11. doi: 10.1186/1471-2164-15-S6-S11 (PMC4239591; doi:10.1186/1471-2164-15-S6-S11)
Supplement: Additional file 1 — 1471-2164-15-S6-S11-S1.pdf. Additional figures and tables omitted from the main paper due to space constraints are presented here. [file 1471-2164-15-S6-S11-S1.pdf]

RESEARCH

Supplementary materials for "BBCA: Improving the scalability of *BEAST using random binning"

Théo Zimmermann, Siavash Mirarab and Tandy Warnow*

*Correspondence:
warnow@illinois.edu
Full list of author information is available at the end of the article

Table 1 List of taxon sampling for the Laurasiatheria datasets.

3-letter code	Species	Common Name
VIC	Vicugna pacos	Alpaca
BOS	Bos taurus	Cow
SUS	Sus scrofa	Pig
EQU	Equus caballus	Horse
CAN	Canis familiaris	Dog
FEL	Felis catus	Cat
TUR	Tursiops truncatus	Dolphin
ERI	Erinaceus europaeus	Hedgehog
PTE	Pteropus vampyrus	Megabat
MYO	Myotis lucifugus	Microbat
SOR	Sorex araneus	Shrew
HOM	Homo sapiens	Human

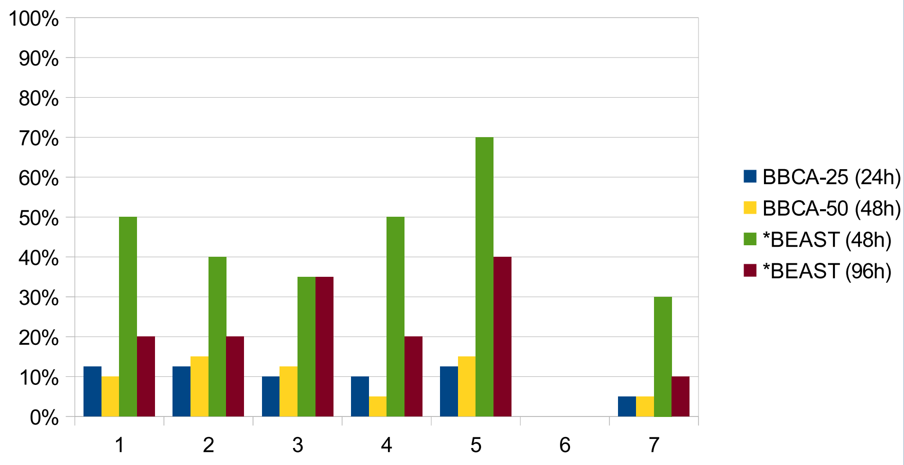

Figure 1 ESS values below 100 on 11 taxon datasets. Proportion of ESS values below 100 when running BBCA with 25-gene bins (blue), BBCA with 50-gene bins (yellow), *BEAST for 48 hours (green) or *BEAST for 96 hours (dark red) on the ten simulated 11-taxon datasets with 100 genes; ESS values above 100 are necessary for convergence. We report these proportions for (1) posterior, (2) prior, (3) likelihood, (4) species.coalescent, (5) species.popSizesLikelihood, (6) speciation.likelihood, (7) species.popMean. Thus, BBCA had converged better than *BEAST, even when the total cumulative runtime was the same.

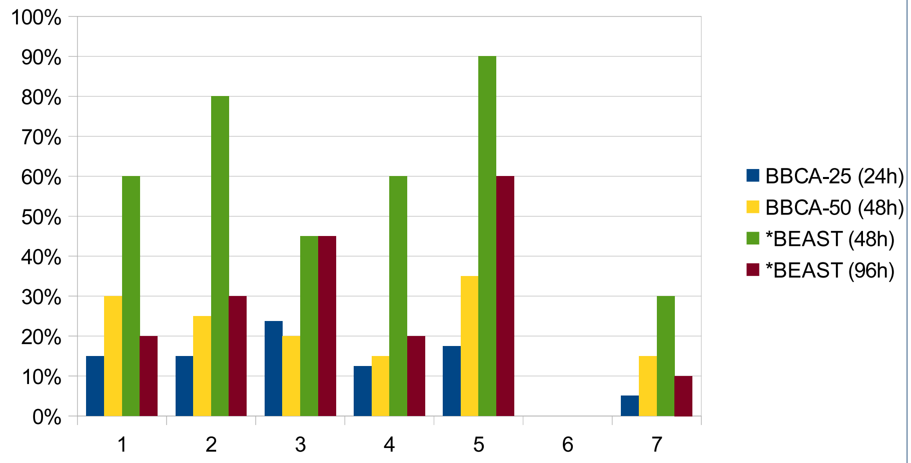

Figure 2 ESS values below 200 on 11 taxon datasets. Proportion of ESS values below 200 when running BBCA with 25-gene bins (blue), BBCA with 50-gene bins (yellow), *BEAST for 48 hours (green) or *BEAST for 96 hours (dark red) on the ten simulated 11-taxon datasets with 100 genes; ESS values above 200 are desirable to assess convergence. We report these proportions for (1) posterior, (2) prior, (3) likelihood, (4) species.coalescent, (5) species.popSizesLikelihood, (6) speciation.likelihood, (7) species.popMean.

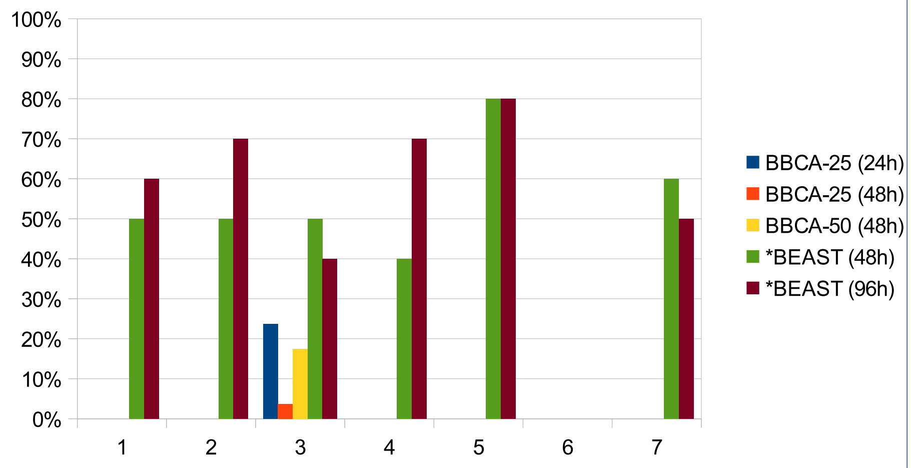

Figure 3 ESS values below 100 on Laurasiatheria 500bp datasets. Proportion of ESS values below 100 when running BBCA with 25-gene bins for 24 hours (blue), BBCA with 25-gene bins for 48 hours (red), BBCA with 50-gene bins for 48 hours (yellow), *BEAST for 48 hours (green), or *BEAST for 96 hours (dark red) on the ten simulated 500bp Laurasiatheria datasets with 100 genes; ESS values above 100 are necessary for convergence. We report these proportions for (1) posterior, (2) prior, (3) likelihood, (4) species.coalescent, (5) species.popSizesLikelihood, (6) speciation.likelihood, (7) species.popMean. Thus, BBCA had converged better than *BEAST, even when the total cumulative runtime was the same. The best convergence on the likelihood statistics was obtained by running BBCA on the smallest bins for the longest time.

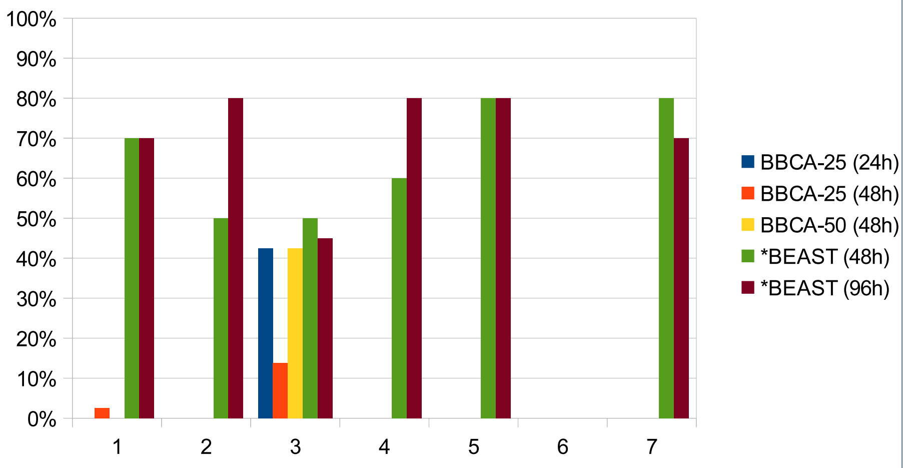

Figure 4 ESS values below 200 on Laurasiatheria 500bp datasets. Proportion of ESS values below 200 when running BBCA with 25-gene bins for 24 hours (blue), BBCA with 25-gene bins for 48 hours (red), BBCA with 50-gene bins for 48 hours (yellow), *BEAST for 48 hours (green), or *BEAST for 96 hours (dark red) on the ten simulated 500bp Laurasiatheria datasets with 100 genes; ESS values above 200 are desirable to assess convergence. We report these proportions for (1) posterior, (2) prior, (3) likelihood, (4) species.coalescent, (5) species.popSizesLikelihood, (6) speciation.likelihood, (7) species.popMean.

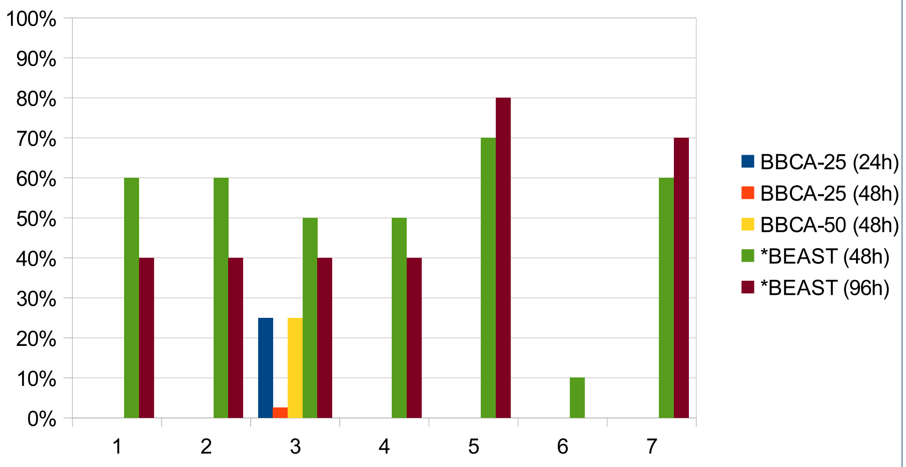

Figure 5 ESS values below 100 on Laurasiatheria 1000bp datasets. Proportion of ESS values below 100 when running BBCA with 25-gene bins for 24 hours (blue), BBCA with 25-gene bins for 48 hours (red), BBCA with 50-gene bins for 48 hours (yellow), *BEAST for 48 hours (green), or *BEAST for 96 hours (dark red) on the ten simulated 1000bp Laurasiatheria datasets with 100 genes; ESS values above 100 are necessary for convergence. We report these proportions for (1) posterior, (2) prior, (3) likelihood, (4) species.coalescent, (5) species.popSizesLikelihood, (6) speciation.likelihood, (7) species.popMean.

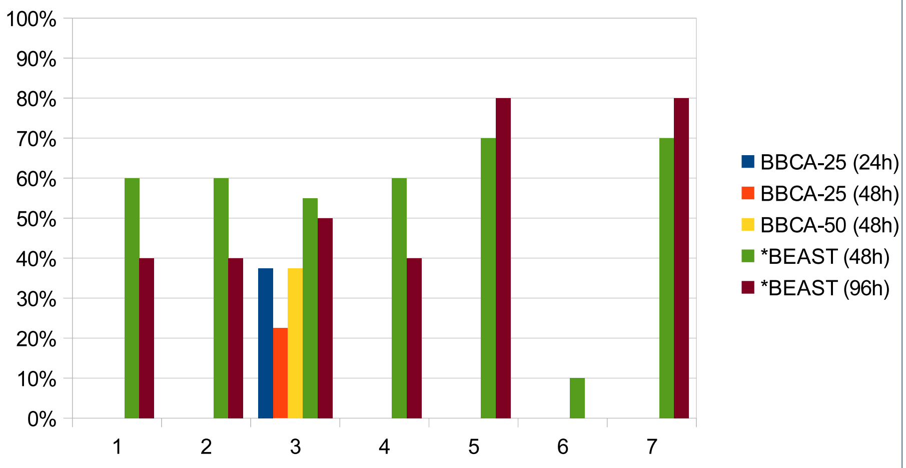

Figure 6 ESS values below 200 on Laurasiatheria 1000bp datasets. Proportion of ESS values below 200 when running BBCA with 25-gene bins for 24 hours (blue), BBCA with 25-gene bins for 48 hours (red), BBCA with 50-gene bins for 48 hours (yellow), *BEAST for 48 hours (green), or *BEAST for 96 hours (dark red) on the ten simulated 1000bp Laurasiatheria datasets with 100 genes; ESS values above 200 are desirable to assess convergence. We report these proportions for (1) posterior, (2) prior, (3) likelihood, (4) species.coalescent, (5) species.popSizesLikelihood, (6) speciation.likelihood, (7) species.popMean.

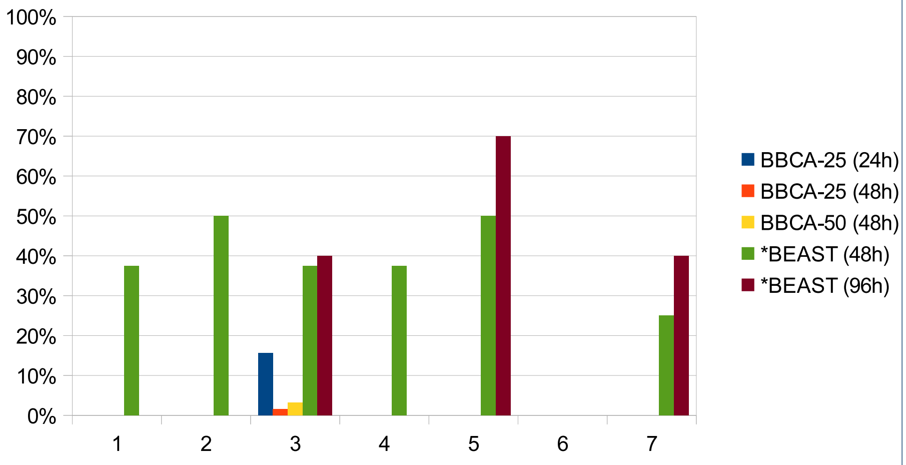

Figure 7 ESS values below 100 on Laurasiatheria 1500bp datasets. Proportion of ESS values below 100 when running BBCA with 25-gene bins for 24 hours (blue), BBCA with 25-gene bins for 48 hours (red), BBCA with 50-gene bins for 48 hours (yellow), *BEAST for 48 hours (green), or *BEAST for 96 hours (dark red) on eight simulated 1500bp Laurasiatheria datasets with 100 genes; ESS values above 100 are necessary for convergence. We report these proportions for (1) posterior, (2) prior, (3) likelihood, (4) species.coalescent, (5) species.popSizesLikelihood, (6) speciation.likelihood, (7) species.popMean.

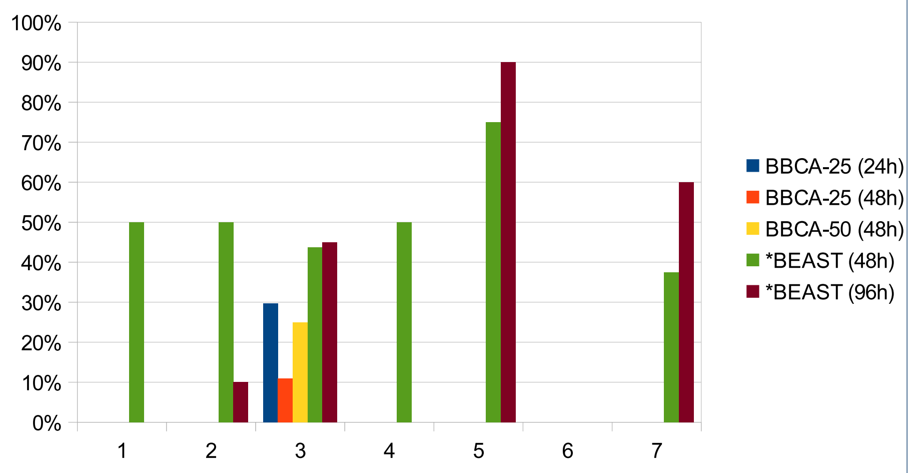

Figure 8 ESS values below 200 on Laurasiatheria 1500bp datasets. Proportion of ESS values below 200 when running BBCA with 25-gene bins for 24 hours (blue), BBCA with 25-gene bins for 48 hours (red), BBCA with 50-gene bins for 48 hours (yellow), *BEAST for 48 hours (green), or *BEAST for 96 hours (dark red) on eight simulated 1500bp Laurasiatheria datasets with 100 genes; ESS values above 200 are desirable to assess convergence. We report these proportions for (1) posterior, (2) prior, (3) likelihood, (4) species.coalescent, (5) species.popSizesLikelihood, (6) speciation.likelihood, (7) species.popMean.

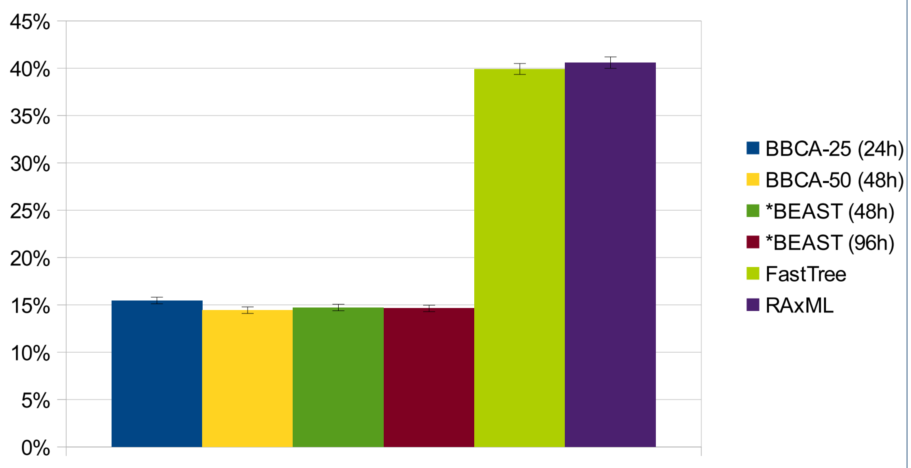

Figure 9 Average gene tree estimation error on the ten simulated 11-taxon datasets with 100 genes. *BEAST and BBCA produce gene trees with essentially the same accuracy (only 15% RF error), while RAxML and FastTree had much higher error rates (40% RF error). Error bars show standard error.

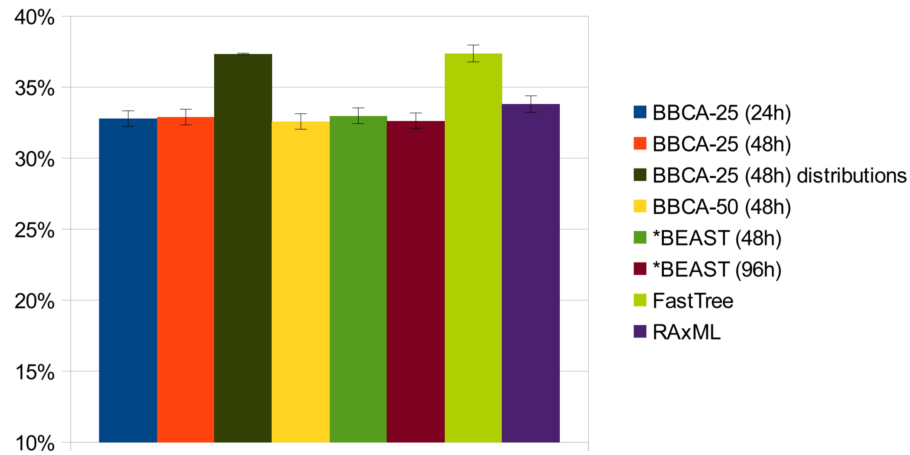

Figure 10 Gene tree error on the ten Laurasiatheria 500bp datasets. Results are reported for BBCA with 25-gene bins and a 24-hour runtime (blue), BBCA with 25-gene bins and a 48-hour runtime (red), distributions of gene trees (rather than single best trees) estimated with BBCA with 25-gene bins and a 48-hour runtime (dark green), BBCA with 50-gene bins and a 48-hour runtime (yellow), *BEAST with a 48-hour runtime (green), *BEAST with a 96-hour runtime (dark red), FastTree (light green) and RAxML (purple). Error bars show standard error.

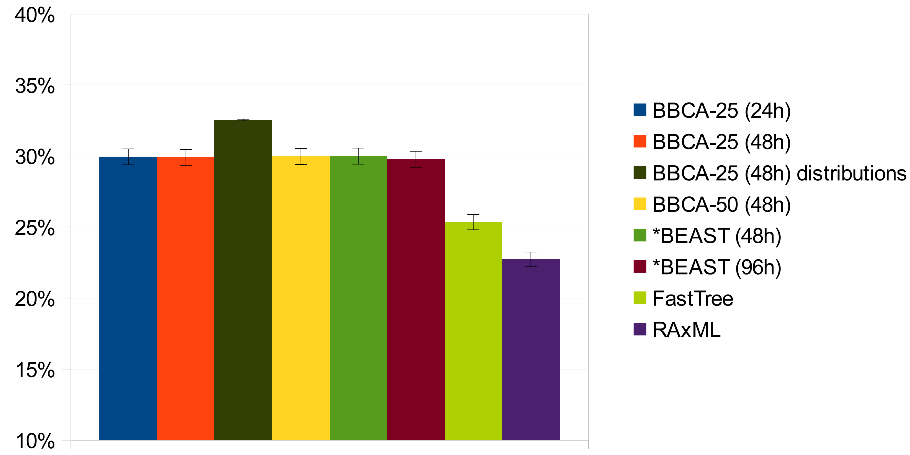

Figure 11 Gene tree error on the ten Laurasiatheria 1000bp datasets. Results are reported for BBCA with 25-gene bins and a 24-hour runtime (blue), BBCA with 25-gene bins and a 48-hour runtime (red), distributions of gene trees (rather than single best trees) estimated with BBCA with 25-gene bins and a 48-hour runtime (dark green), BBCA with 50-gene bins and a 48-hour runtime (yellow), *BEAST with a 48-hour runtime (green), *BEAST with a 96-hour runtime (dark red), FastTree (light green) and RAxML (purple). Error bars show standard error.

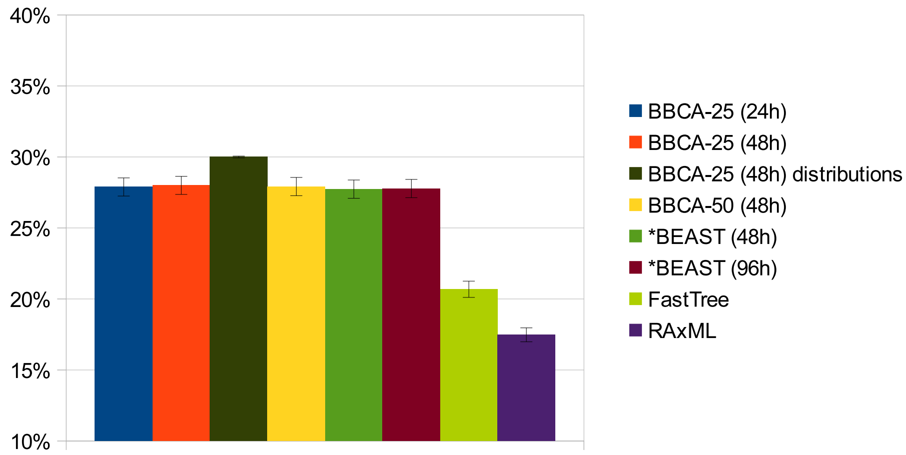

Figure 12 Gene tree error on eight Laurasiatheria 1500bp datasets. Results are reported for BBCA with 25-gene bins and a 24-hour runtime (blue), BBCA with 25-gene bins and a 48-hour runtime (red), distributions of gene trees (rather than single best trees) estimated with BBCA with 25-gene bins and a 48-hour runtime (dark green), BBCA with 50-gene bins and a 48-hour runtime (yellow), *BEAST with a 48-hour runtime (green), *BEAST with a 96-hour runtime (dark red), FastTree (light green) and RAxML (purple). Error bars show standard error.

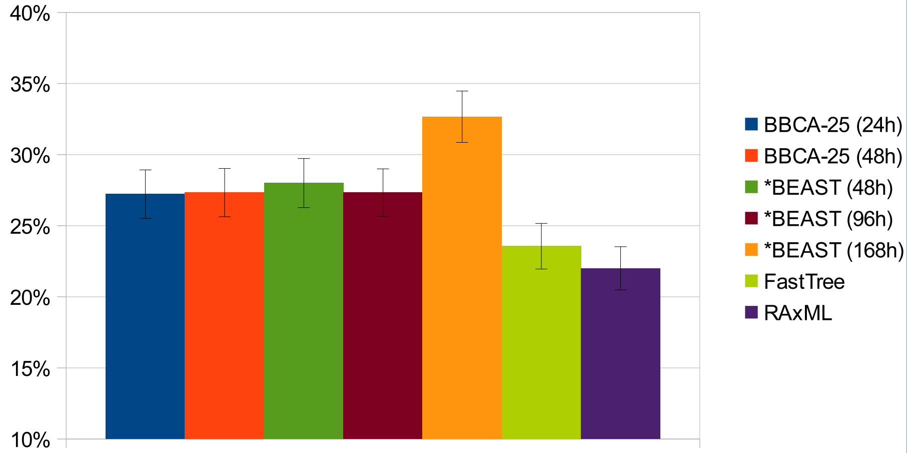

Figure 13 Average gene tree estimation error on one Laurasiatheria 1000bp simulated dataset using BBCA, *BEAST, FastTree and RAxML. BBCA is run with a 24-hour or 48-hour time limit on each 25-gene bin, and *BEAST is run with a 48-hour, 96-hour or 168-hour time limit.

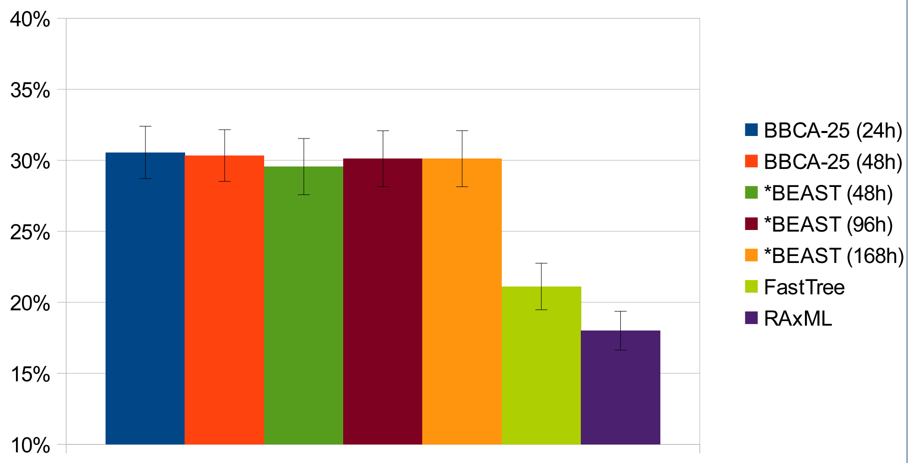

Figure 14 Average gene tree estimation error on one Laurasiatheria 1500bp simulated dataset using BBCA, *BEAST, FastTree and RAxML. BBCA is run with a 24-hour or 48-hour time limit on each 25-gene bin, and *BEAST is run with a 48-hour, 96-hour or 168-hour time limit.

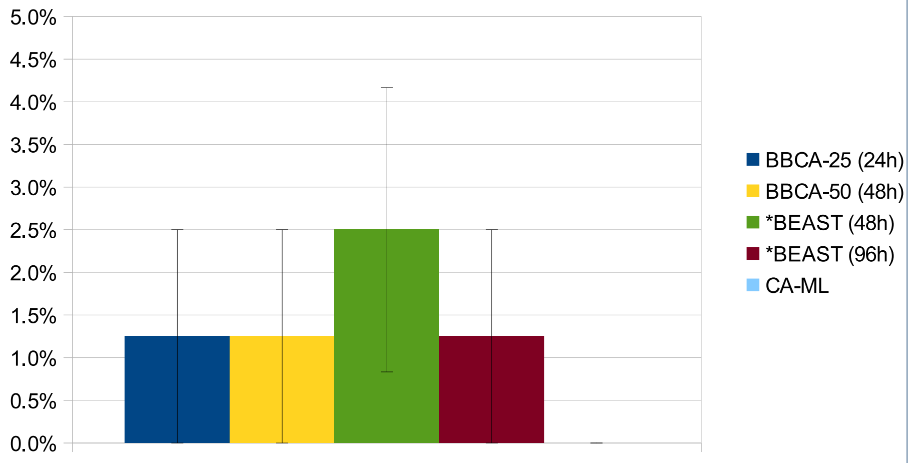

Figure 15 Average species tree estimation error on the ten 11-taxon simulated datasets. Results are reported for BBCA with 25-gene bins (blue), BBCA with 50-gene bins (yellow), *BEAST with a 48-hour runtime (green), *BEAST with a 96-hour runtime (dark red) and concatenation using RAxML (light blue). Both instances of BBCA, as well as *BEAST with a 96-hour runtime, give an average of 1.25% error (i.e., one incorrect branch in one out of 10 replicates). The bar for concatenation using RAxML does not appear because it had 0% error on the 10 replicates. Error bars show standard error.

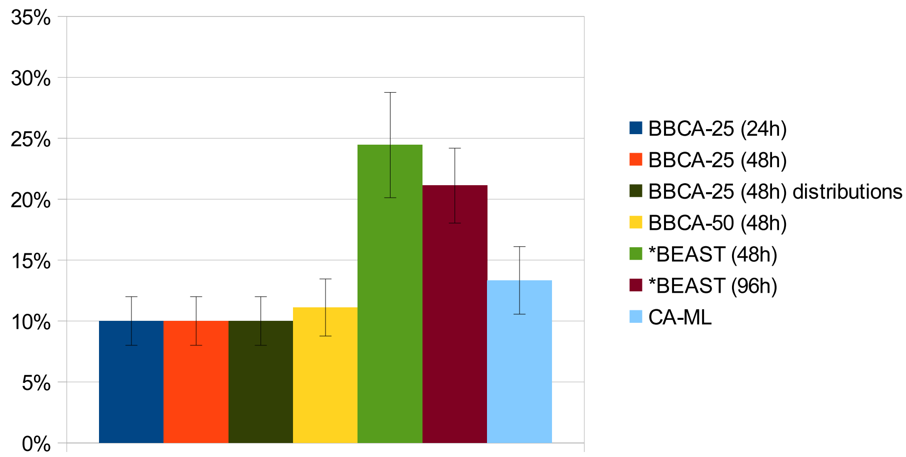

Figure 16 Average species tree estimation error on the ten Laurasiatheria 500bp simulated datasets. Results are reported for BBCA with 25-gene bins and a 24-hour runtime (blue), BBCA with 25-gene bins and a 48-hour runtime (red), BBCA with 25-gene bins and a 48-hour runtime calling MP-EST on distributions of gene trees rather than single best gene trees (dark green), BBCA with 50-gene bins and a 48-hour runtime (yellow), *BEAST with a 48-hour runtime (green), *BEAST with a 96-hour runtime (dark red) and concatenation using RAxML (light blue). Error bars show standard error.

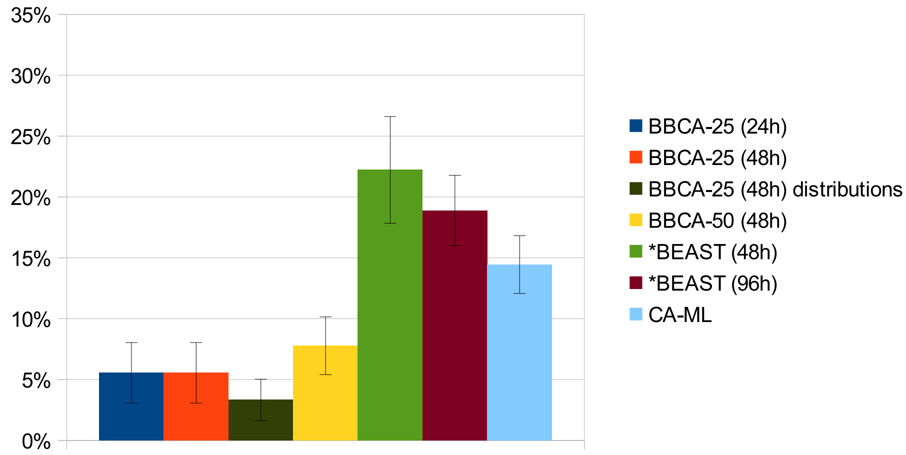

Figure 17 Average species tree estimation error on the ten Laurasiatheria 1000bp simulated datasets. Results are reported for BBCA with 25-gene bins and a 24-hour runtime (blue), BBCA with 25-gene bins and a 48-hour runtime (red), BBCA with 25-gene bins and a 48-hour runtime calling MP-EST on distributions of gene trees rather than single best gene trees (dark green), BBCA with 50-gene bins and a 48-hour runtime (yellow), *BEAST with a 48-hour runtime (green), *BEAST with a 96-hour runtime (dark red) and concatenation using RAxML (light blue). Error bars show standard error.

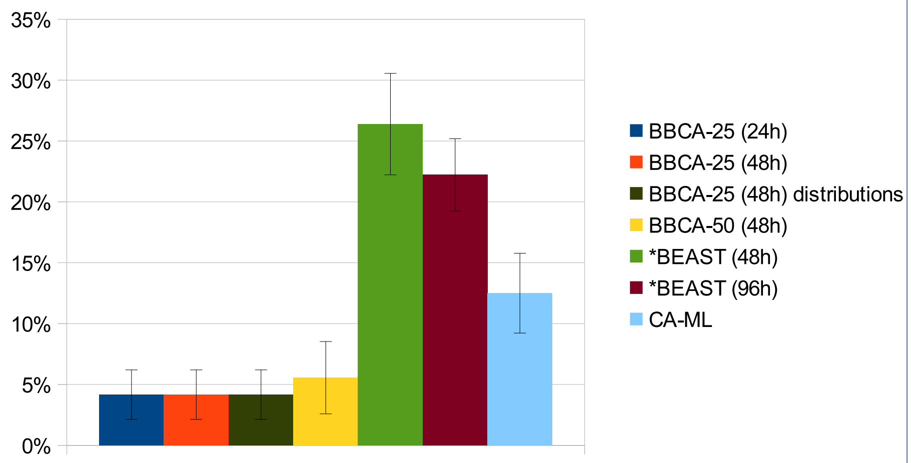

Figure 18 Average species tree estimation error on eight Laurasiatheria 1500bp simulated datasets. Results are reported for BBCA with 25-gene bins and a 24-hour runtime (blue), BBCA with 25-gene bins and a 48-hour runtime (red), BBCA with 25-gene bins and a 48-hour runtime calling MP-EST on distributions of gene trees rather than single best gene trees (dark green), BBCA with 50-gene bins and a 48-hour runtime (yellow), *BEAST with a 48-hour runtime (green), *BEAST with a 96-hour runtime (dark red) and concatenation using RAxML (light blue). Error bars show standard error.
